# Comparative Phytotoxicity of Metallic Elements on Duckweed *Lemna gibba* L. Using Growth- and Chlorophyll Fluorescence Induction-Based Endpoints

**DOI:** 10.3390/plants13020215

**Published:** 2024-01-12

**Authors:** Muhammad Irfan, Ilona Mészáros, Sándor Szabó, Viktor Oláh

**Affiliations:** 1 Department of Botany, Institute of Biology and Ecology, Faculty of Science and Technology, University of Debrecen, Egyetem Square 1, H-4032 Debrecen, Hungary; muhadirfan6167@gmail.com (M.I.); immeszaros@unideb.hu (I.M.); 2 Department of Biology, Institute of Environmental Sciences, University of Nyiregyhaza, H-4401 Nyiregyhaza, Hungary

**Keywords:** duckweed, *Lemna gibba*, phytotoxicity, growth inhibition, chlorophyll fluorescence induction, heavy metal, metalloid

## Abstract

In this study, we exposed a commonly used duckweed species—*Lemna gibba* L.—to twelve environmentally relevant metals and metalloids under laboratory conditions. The phytotoxic effects were evaluated in a multi-well-plate-based experimental setup by means of the chlorophyll fluorescence imaging method. This technique allowed the simultaneous measuring of the growth and photosynthetic parameters in the same samples. The inhibition of relative growth rates (based on frond number and area) and photochemical efficiency (F_v_/F_o_ and Y(II)) were both calculated from the obtained chlorophyll fluorescence images. In the applied test system, growth-inhibition-based phytotoxicity endpoints proved to be more sensitive than chlorophyll-fluorescence-based ones. Frond area growth inhibition was the most responsive parameter with a median EC_50_ of 1.75 mg L^−1^, while F_v_/F_o_, the more responsive chlorophyll-fluorescence-based endpoint, resulted in a 5.34 mg L^−1^ median EC_50_ for the tested metals. Ag (EC_50_ 0.005–1.27 mg L^−1^), Hg (EC_50_ 0.24–4.87 mg L^−1^) and Cu (EC_50_ 0.37–1.86 mg L^−1^) were the most toxic elements among the tested ones, while As(V) (EC_50_ 47.15–132.18 mg L^−1^), Cr(III) (EC_50_ 6.22–19.92 mg L^−1^), Se(VI) (EC_50_ 1.73–10.39 mg L^−1^) and Zn (EC_50_ 3.88–350.56 mg L^−1^) were the least toxic ones. The results highlighted that multi-well-plate-based duckweed phytotoxicity assays may reduce space, time and sample volume requirements compared to the standard duckweed growth inhibition tests. These benefits, however, come with lowered test sensitivity. Our multi-well-plate-based test setup resulted in considerably higher median EC_50_ (3.21 mg L^−1^) for frond-number-based growth inhibition than the 0.683 mg L^−1^ median EC_50_ derived from corresponding data from the literature with standardized *Lemna*-tests. Under strong acute phytotoxicity, frond parts with impaired photochemical functionality may become undetectable by chlorophyll fluorometers. Consequently, the plant parts that are still detectable display a virtually higher average photosynthetic performance, leading to an underestimation of phytotoxicity. Nevertheless, multi-well-plate-based duckweed phytotoxicity assays, combined with chlorophyll fluorescence imaging, offer definite advantages in the rapid screening of large sample series or multiple species/clones. As chlorophyll fluorescence images provide information both on the photochemical performance of the test plants and their morphology, a joint analysis of the two endpoint groups is recommended in multi-well-plate-based duckweed phytotoxicity assays to maximize the information gained from the tests.

## 1. Introduction

**Figure 1:**
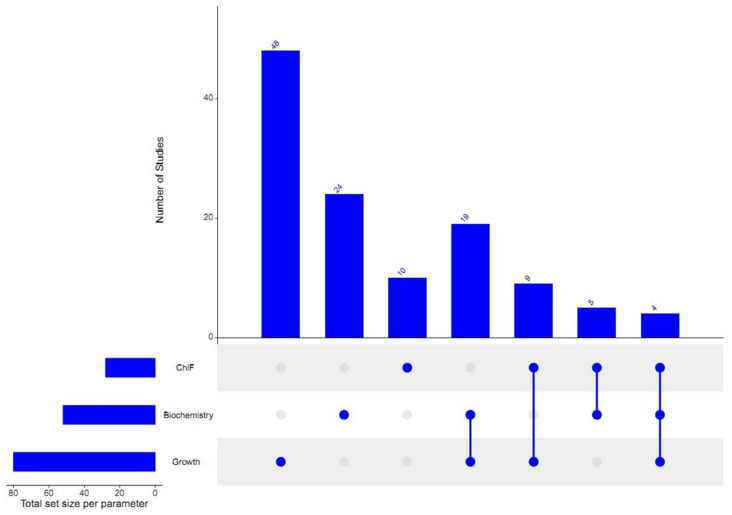
The occurrence of different test endpoints based on growth, biochemical markers and chlorophyll fluorescence induction parameters (ChlF) in duckweed phytotoxicity studies published between 2012 and 2023. The studied species, the parameters within the three endpoint groups and the references used are provided in the Supplementary File S1.

Duckweeds (Lemnaceae) are recommended for ecotoxicological studies as model plants for aquatic macrophytes because of their sensitivity, simple anatomy and fast growth rate [1,2]. Furthermore, duckweeds are also being studied as a source of biomass and biofuel production [3,4,5,6], and as phytoremediating agents [7,8,9,10]. For toxicity testing, *Lemna gibba* and *L. minor* are the recommended species in *Lemna*-tests according to the OECD guidelines [11]. In these standardized duckweed toxicity tests, the effects of environmental stressors are principally measured in terms of growth inhibition (Figure 1). Growth inhibition can be determined based on changes in frond number, area and the fresh or dry weight of the cultures, respectively [11,12]. Furthermore, toxicological studies also incorporated additional test endpoints, such as chlorophyll content [10,13,14], enzyme activities [15,16], colony size [17,18] and root length [19,20]. More recently, as an emerging non-invasive technique, the chlorophyll fluorescence (ChlF) induction method has also been increasingly utilized in various plant- and algae-based ecotoxicological studies [21,22,23].

ChlF-based indicators are proxies for photosynthetic processes. Thus, the method offers high sensitivity in terms of both exposure duration and toxicant concentration [24]. Certain types of imaging chlorophyll fluorometers, such as the Maxi Imaging-PAM (Heinz Walz GmbH, Effeltrich, Germany) and FluorCam (Photon Systems Instruments, Brno, Czech Republic), were designed to suit phytotoxicological applications, e.g., by fitting the dimensions of multi-well plates and offering specific setting options for toxicity assays. However, the availability of a wide range of ChlF parameters, coupled with the selective application of only a few ones by different studies, makes data comparisons difficult [24]. Moreover, further research is needed to comparatively evaluate ChlF-based indicators with jointly measured growth responses [25].

The OECD duckweed toxicity tests are conducted for seven days, and use a minimum of 100 mL growth medium [11]. Large sample series or the need for rapid risk assessment, however, may challenge this standardized protocol. Recent studies have adapted duckweed tests in multi-well plates (usually 6-, 12- or 24-well plates) using a smaller volume of test medium for 3–7 days [19,24,26,27,28]. Such small-scale, multi-well-plate-based experiments can be managed in a limited space and with fewer resources, promising a potential alternative to standard toxicity testing [29,30,31]. So far, however, most multi-well-plate-based studies with duckweeds have focused on small sets of toxicants and/or on narrow concentration ranges. Consequently, a comprehensive evaluation of the suitability and limitations of such experimental set-ups by involving a wider range of toxicants is still needed. Similarly, further research is inevitable on the broader applicability of ChlF-based endpoints in phytotoxicology [24]. In this regard, ChlF imaging is of special interest as this technique provides spatially resolved information. By bearing in mind the potential of joint morphometric and photosynthetic analyses, this method offers a high throughput multi-approach tool to study plant responses [32,33]. The interchangeability of morphometric and photosynthetic responses in the lower and higher extremes of phytotoxicity, however, is yet to be resolved. Duckweeds are particularly suitable for such comparative analyses as they easily fit into multi-well plates and their nearly two-dimensional body-plan is optimal for image analysis. To address the above questions, this study aimed to (i) provide a comprehensive phytotoxicity dataset for *L. gibba* gained in a multi-well-plate-based setup for 12 environmentally relevant metals and metalloids, and (ii) compare the responsivity of growth-based and chlorophyll-fluorescence-based phytotoxicity endpoints to the tested elements by using the same chlorophyll fluorescence images.

## 2. Materials and Methods

### 2.1. Test Materials and Experimental Design

Axenic *Lemna gibba* L. cultures (clone #UD0101, originally isolated in E-Hungary) maintained at the Department of Botany, University of Debrecen, Hungary, were used for this study. The stock cultures were initiated with 6–8 healthy colonies, and grown in 300 mL conical flasks with 100 mL sterile modified Steinberg medium for 7 days prior to the tests [24]. A total of 12 environmentally relevant metals and metalloids (including different oxidation states) were tested in a series of at least eight nominal concentrations. The applied concentration ranges were based on pilot experiments, and the series was designed to cover approximately three magnitudes by doubling the previous concentration at every step. For Ag, Cr(VI) and Se(VI), the pilot experiments indicated highly divergent responses for different endpoints. Thus, we covered wider concentration ranges with additional treatment levels for these metals. The tested elements and concentrations were as follows: silver (AgNO_3_; 0.76 ng L^−1^–0.25 mg L^−1^), arsenite (i.e., As(III) as NaAsO_2_; 0.078–10 mg L^−1^), arsenate (i.e., As(V) as Na_2_HAsO_4_; 0.78–100 mg L^−1^), cadmium (CdCl_2_; 0.078–10 mg L^−1^), chromite (i.e., Cr(III) as KCr(SO_4_)_2_ ·12H_2_O; 0.78–100 mg L^−1^), chromate (i.e., Cr(VI) as K_2_CrO_4_; 0.0012–10 mg L^−1^), copper (CuSO_4_·5H_2_O; 0.078–10 mg L^−1^), mercury (HgCl_2_; 0.078–10 mg L^−1^), nickel (NiSO_4_·7H_2_O; 0.078–10 mg L^−1^), selenite (Se(IV) as Na_2_SeO_3_; 0.078–10 mg L^−1^), selenate (i.e., Se(VI) as Na_2_SeO_4_·10 H_2_O; 0.002–10 mg L^−1^) and zinc (ZnSO_4_·7H_2_O; 0.78–100 mg L^−1^). For the treatments, standard 12-well tissue culture plates were used. Each experimental series included four parallel control wells with 4–4 mL of pure Steinberg medium. The treatment wells contained 4 mL Steinberg medium spiked with the respective toxicant concentrations in quadruplicates. A single colony of 3–4 healthy fronds was transferred into each well as a starting inoculum. The test plants were then treated for three days (72 ± 2 h) under continuous white irradiation (PPFD: 60 ± 10 µE m^−2^ s^−1^), at a temperature of 22 ± 2 °C [10]. All experiments with respect to the toxicant and concentration range were repeated twice following the same protocol and under identical ambient conditions.

### 2.2. In Vivo Chlorophyll Fluorescence Induction Measurements

The photosynthetic parameters were measured by means of the in vivo chlorophyll fluorescence imaging method. A Maxi Imaging-PAM chlorophyll fluorometer was used, equipped with a blue-colored LED imaging unit (with a peak intensity at 450 nm) and mounted with an IMAG-K6 digital camera (Heinz Walz GmbH, Effeltrich, Germany). The unit was operated via ImagingWin v2.47 software (Heinz Walz GmbH, Effeltrich, Germany). On the 3rd day of the experiments, we took ChlF images of plants in each plate according to the following protocol. Firstly, the treated plants in multi-well plates were taken out from the culturing room and quickly placed into the measuring chamber of the instrument. Then, the plants were illuminated by a continuous actinic light (450 nm, 81 µmol s^−1^ m^−2^) for 60 s to maintain their light-acclimated state. After 60 s of actinic illumination, while still irradiating the plants, we measured the steady-state ChlF yield (F_s_) and then applied a strong saturation light pulse (~4000 µE m^−2^ s^−1^) to determine the maximum ChlF yield (F_m_′). As a second step, the plants were dark-adapted for 20 min, allowing them to reach fully oxidized state of PSII. After dark adaptation, we placed the plates back in the measuring chamber, and applied a weak, non-inductive measuring light (intensity: 2, frequency: 1) followed by a saturating pulse to measure ground (F_o_) and maximum ChlF yields (F_m_) in the dark-adapted plants, respectively. The basic ChlF parameters of dark-adapted and light-adapted plant samples were used for calculating the following proxies of photosynthetic efficiency averaged on a well basis [34,35]:

F_v_/F_o_ = (F_m_ − F_o_)/F_o_

Y(II) = ∆F/F_m_′ ; where ∆F = F_m_′ − F_s_

F_v_/F_o_ is an analogue of the widely used F_v_/F_m_—i.e., the maximal quantum yield of PSII in the dark-adapted state. This was chosen because of its higher responsiveness compared to F_v_/F_m_ [24]. Y(II) is proportional to the actual quantum yield of PSII in a light-acclimated state under the applied ambient conditions [36].

### 2.3. Measurement of Growth Inhibition

We obtained relative growth rates (RGRs) of cultures using the chlorophyll fluorescence images exported from the ImagingWin v2.47 software (Heinz Walz GmbH, Germany). For this purpose, the F_m_ images of plants taken on the 0th and 3rd day of the experiments were exported to JPEG files with a 640 × 480 pixel resolution. ImageJ 1.54d software was used for the measurement of total area and frond number of the cultures [37]. For determining plant area, the background and roots were removed on the basis of Hue, Saturation and Brightness by means of the ‘Threshold Colour’ plugin [38]. Then, the filtered images were converted to binary, and the plant area in each well was calculated via the ‘Analyze Particles’ function. To visually count fronds, the ‘cell counter’ plugin was applied. We calculated the relative growth rates of cultures with respect to both the plant area (RGR_area_) and the frond number (RGR_frond_) according to the following formula [11]:

RGR_X_ = ln (X_f_ − X_i_)/(t_f_ − t_i_)

where,

X = area or number of fronds;
X_i_ = initial value of the respective growth parameter;
X_f_ = final value of the respective growth parameter;
t_i_ = starting day of treatments (i.e., 0);
t_f_ = final day of treatments (i.e., 3).

### 2.4. Data Analysis and Statistics

Experimental data were pooled from the two repeated experiments with four internal parallel replicates with respect to each metal or metalloid. Thus, an overall sample size of n = 8 for every concentration was analyzed. In order to analyze the concentration–response relationships, three-parameter log-logistic models (LL.3) were fitted by means of the ‘drc’ package [39] in RStudio [40]. The suitability of the fitted models was first visually checked and then tested through the ‘lack of fit’ test (‘modelFit’ function) and ‘Pseudo-R2’ (‘cor’ function). The toxicity of metals and metalloids and the sensitivity of the analyzed test endpoints were characterized by the calculated effective concentrations (EC) resulting in a 20% (EC_20_) and 50% (EC_50_) inhibition of the respective endpoint. For that, the ‘ED’ function of the ‘drc’ package was used. A correlation matrix (Spearman’s correlation) of the calculated effective concentrations was constructed using the ‘corrplot’ package [41] in RStudio. Pairwise comparisons (paired sample Wilcoxon signed ranks test) of the calculated effective concentrations were performed by means of OriginPro 2016 (version b9.3.226; Academic, Northampton, MA, USA).

## 3. Results and Discussion

### 3.1. Growth-Inhibition-Based Endpoints

**Figure 2:**
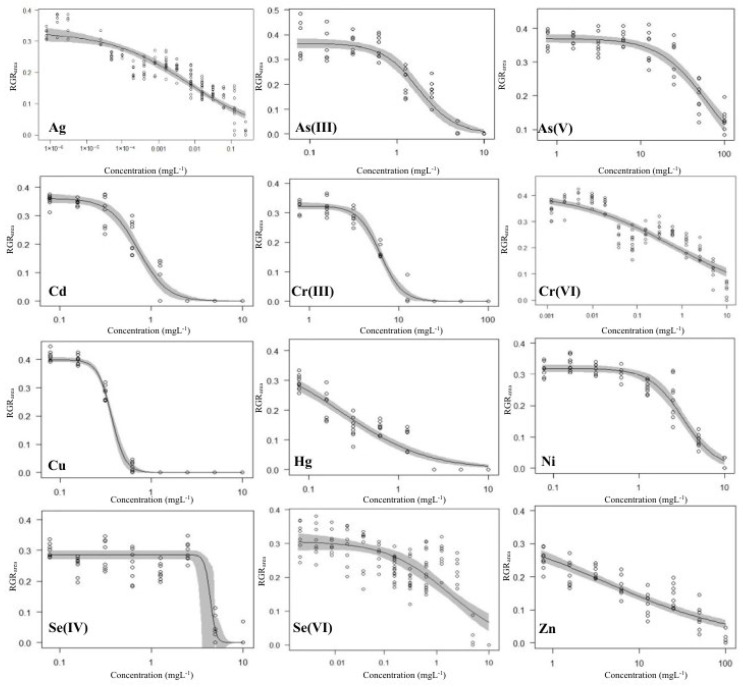
The 3-parameter log-logistic concentration–response model fittings of RGR_area_ for the tested metals and metalloids. Circles denote individual measurements; solid lines and shaded areas denote the fitted models and the corresponding standard errors of estimates.

The average doubling time for the control cultures of *L. gibba* was 1.90 ± 0.24 days in terms of frond area, fulfilling the validity requirements of the OECD [11] guidelines (<2.5 days). Similarly, in terms of frond number, a doubling time of 1.80 ± 0.22 days was recorded by the end of the 3-day-long tests. The corresponding average growth rates for frond area and frond number were 0.37 ± 0.04 and 0.39 ± 0.05, respectively. The growth inhibitory effects of metals and metalloids in higher concentrations were clearly developed by the end of the 3 days of exposure (Figure 2, further plots for RGR_frond_, F_v_/F_o_ and Y(II) are provided in Figures S5–S7). Based on the calculated EC_50_ values (Supplementary Table S1), the order of phytotoxic potential of the tested elements and oxidation forms was as follows:

RGR_frond_: Ag > Cu > Hg > Cd > As(III) > Ni > Cr(VI) > Se(IV) > Se(VI) > Cr(III) > Zn > As(V)

RGR_area_: Ag > Hg > Cu > Cr(VI) > Cd > Se(VI) > As(III) > Ni > Zn > Se(IV) > Cr(III) > As(V)

**Figure 3:**
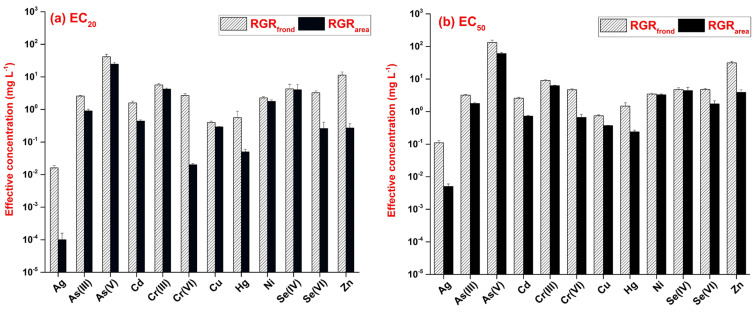
Effective concentrations of the 12 tested metals and metalloids resulting in (**a**) 20% (EC_20_), and (**b**) 50% inhibition (EC_50_) in the frond number—(RGR_frond_, striped bars) and frond-area-based relative growth rates (RGR_area_, black bars). The concentrations and standard errors of estimates (error bars) were calculated based on the fitted 3-parameter log-logistic models. Note the logarithmic scale of the y-axis.

The calculated effective concentrations for different metals were spread over a wide concentration range (Figure 3). In addition, the paired sample Wilcoxon signed ranks tests showed considerably higher responsivity of RGR_area_ to the applied metals and metalloids than that of RGR_frond_, with *p* < 0.001 for both EC_20_ and EC_50_.

Out of the 12 tested toxicants, Ag proved to be the most toxic with the lowest effective concentrations for both RGR_area_ and RGR_frond_. Ag was associated with the induction of oxidative stress and cellular injury in *L. minor* [42]. Additional toxic effects of Ag were also observed in the form of biomass reduction, root abscission and colony disintegration in *Spirodela polyrhiza* [43]. Hg was the second most toxic element among those tested, followed by Cu and Cd (Figure 3). Hg induces oxidative damage, a reduction in the chlorophyll content, DNA damage and, ultimately, cell death [44]. Cu and Cd also affect the photosynthetic pigments and impair the antioxidant defense in duckweeds [45]. On the other extreme of the phytotoxicity spectrum, As(V) and Cr(III) resulted in the highest effective concentrations under the applied experimental conditions. As(V) did not even result in a 50% inhibition in RGR_frond_ within the applied concentration range (Supplementary Table S1 and Figure S5). Zn showed low toxicity to RGR_frond_, while it had intermediate growth inhibition on RGR_area_ compared to other applied toxicants. The different sensitivities of the two endpoints suggested a stress-induced morphogenic response [46], where the inhibiting effect on frond production built up slower along with increasing Zn concentrations, but the newly produced fronds were smaller in size due to restricted elongation.

Our results were comparable to the previously published data in the literature. As(V) was the least toxic based on growth inhibition (i.e., frond number, fresh weight, dry weight) and photosynthetic pigment contents in a previous comparative study on *L. minor* exposed to ten heavy metals [47]. The effective concentrations for RGR_frond_ in *L. gibba* were comparable to the respective values in *L. minor* for Ag, As(III) and Cu [47,48]. Zn was also found to be moderately toxic to duckweeds, up to 10 mg L^−1^ in previous reports [49,50]. The EC_50_ values for Cu from our multi-well-plate-based experiments were also comparable to those ones reported by Khellaf and Zerdaoui [51] using the standard OECD protocol with *L. gibba*. In terms of the 9 commonly analyzed metals, however, our data for *L. gibba* indicated lower sensitivity compared to the previously reported data for *L. minor* by Naumann et al. [47] in ISO [12] standard tests (Table 1). The results for *L. gibba* (current study) and *L. minor* [47] showed a weak correlation for EC_20_ (Spearman’s ρ = 0.55, *p* = 0.125), while the calculated EC_50_ data correlated more strongly (Spearman’s ρ = 0.78, *p* = 0.012). Similarly, the RGR_frond_ and RGR_area_ EC_50_ values in the present study were considerably higher when compared to those previously obtained in our lab using the OECD [11] protocol. Those earlier experiments were performed with *S. polyrhiza* (UD0401 clone) and provided several times lower EC_50_ for RGR_area_ in treatments with Ni and Cr(VI) (0.184 and 0.188 mg L^−1^ [52]), Cd (0.104 mg L^−1^ [18]), Hg (0.137 mg L^−1^ [53]) and As(III) and As(V) (1.33 and 25.04 mg L^−1^, Hepp et al., unpublished data). Ni also proved to be three–four times less toxic in our multi-well-plate-based setup with *L. gibba* compared to data reported for the same species following the OECD protocol [51]. Besides species-specific metal tolerance, these differences were most probably due to the shorter exposure duration and lower dose (i.e., toxicant-to-biomass ratio) in our setup.

In agreement with previous studies, the frond-area-based growth rate showed greater sensitivity to metal-induced inhibition than the frond-number-based one [54,55]. This fact can be explained by the different nature of the two growth parameters. Frond area is a continuous measure while frond number is a quantile one. Daughter fronds may develop to smaller sizes under stress and contribute proportionally less to the total frond area than to the frond number in cultures. This results in stronger growth inhibition, with lower EC values for the former parameter. Additionally, in standard duckweed tests, photos for area measurements are usually taken in the visible spectral range, and can include both healthy and chlorotic spots into the measured frond area. ChlF images, on the other hand, show only the photosynthetically active areas of fronds, while chlorotic regions with a weak ChlF signal can remain undetected. This way, the observed area can drop considerably even when frond number stays seemingly high. Hence, ChlF-based area measurement might have also contributed to the different sensitivities of the two growth rates. 

### 3.2. Chlorophyll-Fluorescence-Induction-Based Endpoints

For the two analyzed ChlF-based endpoints, the respective EC_50_ values (Table S5) indicated the following order of phytotoxicity amongst the tested metals and metalloids:

F_v_/F_o_: Cr(VI) > Cu > Ag > As(III) > Cd > Hg > Ni > Se(IV) > Se(VI) > Cr(III) > Zn > As(V)

Y(II): Ag > Cr(VI) > Cu > As(III) > Cd > Hg > Ni > Se(VI) > Se(IV)> Cr(III) > As(V) > Zn

**Figure 4:**
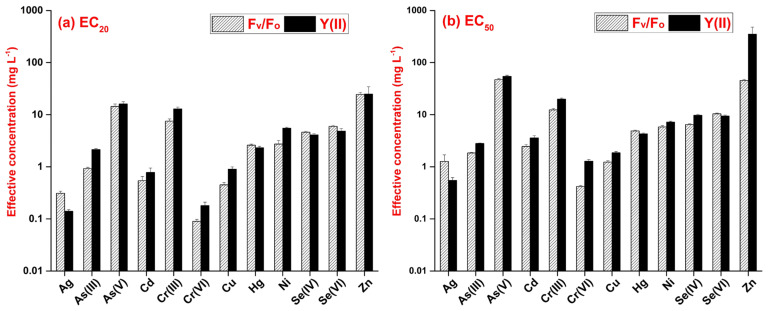
Effective concentrations of the 12 tested metals and metalloids resulting in (**a**) 20% (EC_20_), and (**b**) 50% inhibition (EC_50_) in F_v_/F_o_ (striped bars) and Y(II) (black bars). The concentrations and standard errors of estimates (error bars) were calculated based on the fitted 3-parameter log-logistic models. Note the logarithmic scale of the y-axis.

The paired sample Wilcoxon signed ranks test confirmed that the calculated EC_20_ for the two ChlF parameters did not differ significantly (medians: 2.665 and 3.21 mg L^−1^ for F_v_/F_o_ and for Y(II), respectively, *p* = 0.176). However, the EC_50_ values indicated a significantly higher sensitivity of F_v_/F_o_ to the tested metals and metalloids (medians: 5.34 and 5.755 mg L^−1^ for F_v_/F_o_ and Y(II), respectively, *p* = 0.021). Y(II) was more responsive to Ag, Hg and Se(VI), but F_v_/F_o_ resulted in lower EC values for the rest of the tested metals (Figure 4). For Ag, Se(VI) and Zn, however, we could only calculate the extrapolated effective concentrations, which exceeded the applied concentration ranges (Supplementary Table S1). In that regard, Ag was so toxic to *L. gibba* that the plants lost their viability even before reaching 20% inhibition in either ChlF parameter. For Zn, on the other hand, we could only calculate an unrealistically high (~350 mg L^−1^) EC_50_ in case of Y(II) due to this extrapolation. Contrastingly, EC_50_ for F_v_/F_o_ stayed in the applied concentration range. For Se(VI), the extrapolated EC_50_ for F_v_/F_o_ was only slightly above the applied concentration range.

**Figure 5:**
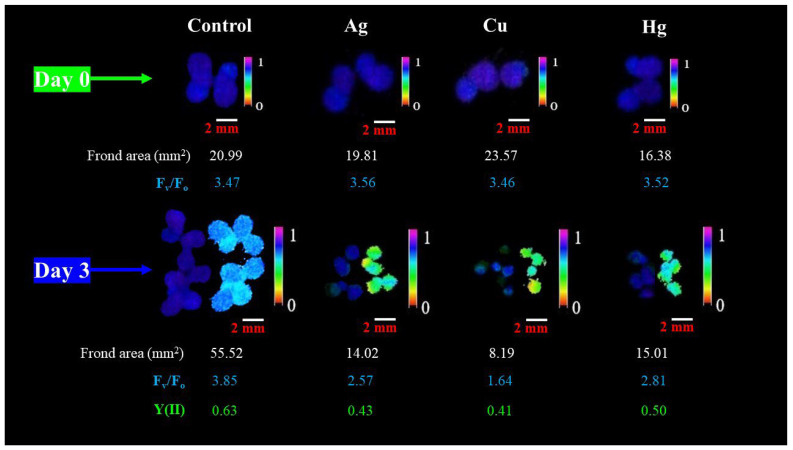
Chlorophyll fluorescence images of the same control and treated *L. gibba* colonies at the beginning (day 0) and final day (day 3) of tests. The treated plants showed significant loss in the detectable frond area due to chlorosis, while the residual, photosynthetically active frond parts still maintained >50% of control F_v_/F_o_ and Y(II). Ag, Cu and Hg denote treatments with 3.125 µg L^−1^ Ag, 1.25 mg L^−1^ Cu and 2.5 mg L^−1^ Hg, respectively. The upper row shows F_v_/F_m_ while the lower row represents F_v_/F_m_ (left side) and Y(II) (right side) for the respective treatments.

When we compared the more sensitive growth-based endpoint (RGR_area_) to the more sensitive ChlF-based one (F_v_/F_o_), the paired sample Wilcoxon signed ranks test confirmed a significantly higher responsivity of growth to metals and metalloids. The difference was seven-fold in the case of the calculated EC_20_ values (medians: 0.365 and 2.665 mg L^−1^ for RGR_area_ and F_v_/F_o_, respectively, *p* = 0.042) and three-fold for the calculated EC_50_ values (medians: 1.75 and 5.34 mg L^−1^ for RGR_area_ and F_v_/F_o_, respectively, *p* = 0.042). As an exception, F_v_/F_o_ proved to be more sensitive than RGR_area_ in the case of As(V) and Cr(VI). We also observed that the comparability of the two parameters mainly decreased in the case of extremely high or low toxicity (e.g., Ag, Se(VI) and Zn). The reason for such diverging responses might be that the higher the toxicant concentration, the greater the loss of apparent frond area due to chlorotic regions. Thus, using ChlF-based F_m_ images for measuring growth inhibition might result in overestimation. As a limitation of the ChlF imaging method in duckweed phytotoxicity tests, a threshold was set for the images at a minimal fluorescence level in order to reduce background noise [56]. In some cases, further pixels of severely impacted frond parts were also non-detectable under the saturating light pulse. As a result, only those frond regions that were still detectable were considered for calculating F_v_/F_o_ and Y(II). Thus, a virtually higher functionality of photochemical efficiency was maintained due to excluding the most affected frond parts (Figure 5). 

A common stress response in duckweeds is the premature detachment of fronds from the parent colony [57]. In our study, this separation was observed in the presence of highly toxic elements (e.g., Ag and Hg) and under the highest concentration treatments. Most of the prematurely separated fronds did not produce further daughter fronds, thus reducing the overall relative growth rate. Nevertheless, these separated small-sized fronds still maintained a certain degree of photosynthetic activity (Figure 5). Combined with the above-described bias by chlorotic spots, these results point to the importance of cautious interpretation of ChlF-based endpoints in duckweed tests.

**Figure 6:**
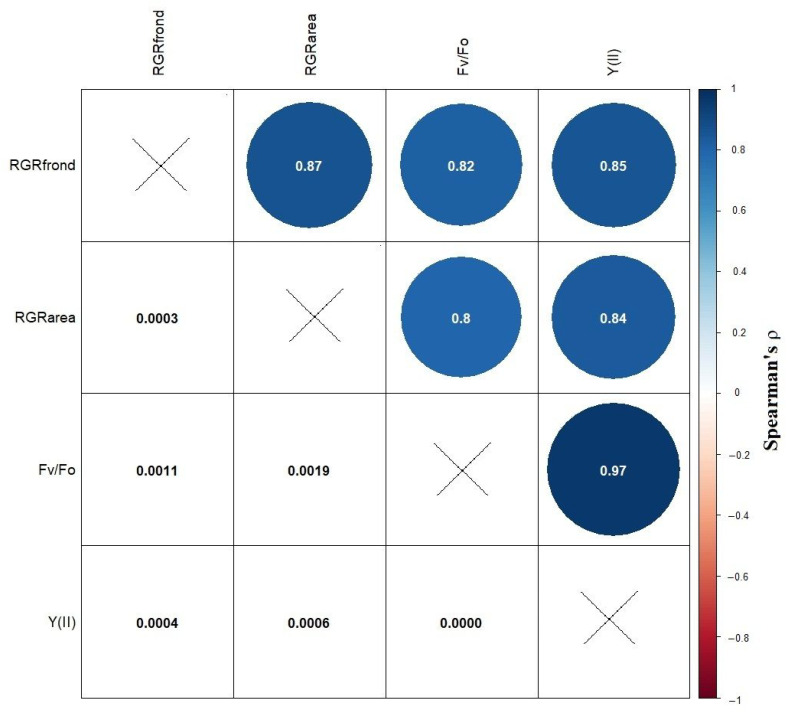
Correlation matrix based on Spearman’s ρ (upper triangle) and the corresponding *p*-values (lower triangle) for the calculated EC_50_ values for the growth- and ChlF-based endpoints.

Even if growth- and ChlF-based endpoints showed diverging sensitivities in the higher and lower extremes of phytotoxicity, their overall correlation indicated strong interdependence (Figure 6). In this regard, our results were consistent with previous data from the literature reporting that a decline in Y(II) strongly correlated with growth inhibition in duckweeds treated with common PSII inhibitor herbicides (e.g., atrazine and diuron) [58]. Similarly, ChlF-derived endpoints proved to be very sensitive in *L. minor* in response to Cu toxicity [59] and also correlated well with changes in fresh weight under bisphenol A exposure [60]. When the applied toxicants (perfluorooctanoic acid and dimethyl phthalate) did not result in growth inhibition, however, ChlF-based endpoints were also unresponsive [33,61]. Our results thus highlight that ChlF-based endpoints can characterize phytotoxicity with certain limitations. More importantly, this non-invasive method can provide functional insight into the modes of action behind toxic effects. Since chlorophyll fluorescence images bear the possibility of extracting information on both the morphology (i.e., growth) and the functional state (i.e., photosynthesis), the joint measurement of both endpoint groups is recommended in duckweed phytotoxicity assays to maximize the information gained from the tests.

## 4. Conclusions

Our results revealed that small-scale, multi-well-plate-based phytotoxicity tests with duckweeds offer definite advantages by reducing space, time and sample volume requirements, but at the expense of lower sensitivity. The order of phytotoxic potential amongst the tested elements was comparable to previous studies using OECD and ISO standard duckweed tests, but the calculated effective concentrations were higher than those in the standardized tests. In addition, when comparing the calculated effective concentrations, we found noticeable differences in the responsivity of the tested endpoints. The results pointed out that chlorophyll-fluorescence-induction-based phytotoxicity endpoints—at least in the case of duckweeds—cannot fully replace the growth-inhibition-based ones. The method inevitably underestimates the photosynthesis-inhibiting effects under strong acute phytotoxicity due to the exclusion of chlorotic frond parts. Nevertheless, chlorophyll fluorescence imaging is valuable in providing a non-invasive tool to jointly analyze duckweed growth and photosynthetic responses in phytotoxicity tests. Thus, the application of multi-well-plate-based duckweed phytotoxicity assays, combined with chlorophyll fluorescence imaging, can facilitate the screening of large sample series or multiple duckweed species/clones.

## Figures and Tables

**Table 1:** Comparison of the sensitivity in the multi-well-plate-based setup (current study) with previously reported effective concentrations obtained from ISO-standard [12] tests by Naumann et al. [47]. The paired sample Wilcoxon signed ranks tests were performed on the calculated 20% (EC_20_) and 50% (EC_50_) effective concentrations for RGR_frond_ using the following 9 common metals in the two studies: Ag, As(III), As(V), Cd, Cr(VI), Cu, Hg, Ni and Zn.

	Median Effective Concentration (RGR_frond_)			
	Current Study (*L. gibba*)	Naumann et al. [47] (*L. minor*)	W	*p*
EC_20_	2.27	0.086	2	0.023
EC_50_	3.21	0.683	3	0.020

## Data Availability

The datasets used in the present study are available from the corresponding author on reasonable request.

## Supplementary Materials

The following supporting information can be downloaded at https://www.mdpi.com/article/10.3390/plants13020215/s1, File S1: metadata for Figures S1–S4 (main text); Figure S5: 3-parameter log-logistic concentration–response model fittings of RGR_frond_; Figure S6: 3-parameter log-logistic concentration–response model fittings of F_v_/F_o_; Figure S7: 3-parameter log-logistic concentration–response model fittings of Y(II); and Table S1: The results of the 3-parameter log-logistic model fittings with calculated EC_20_ and EC_50_ values for the studied endpoints. References [62,63,64,65,66,67,68,69,70,71,72,73,74,75,76,77,78,79,80,81,82,83,84,85,86,87,88,89,90,91,92,93,94,95,96,97,98,99,100,101,102,103,104,105,106,107,108,109,110,111] are cited in the supplementary materials.
