# Supplementary material for: Comparative Phytotoxicity of Metallic Elements on Duckweed Lemna gibba L. Using Growth- and Chlorophyll Fluorescence Induction-Based Endpoints
Source: Plants (Basel). 2024 Jan 12;13(2):215. doi: 10.3390/plants13020215 (PMC10821045; doi:10.3390/plants13020215)

Figure S5. 3-parameter log-logistic concentration-response model fittings of RGR_{frond} for the tested metals and metalloids. Circles denote individual measurements, solid lines and shaded areas denote the fitted models and the corresponding standard errors of estimates.

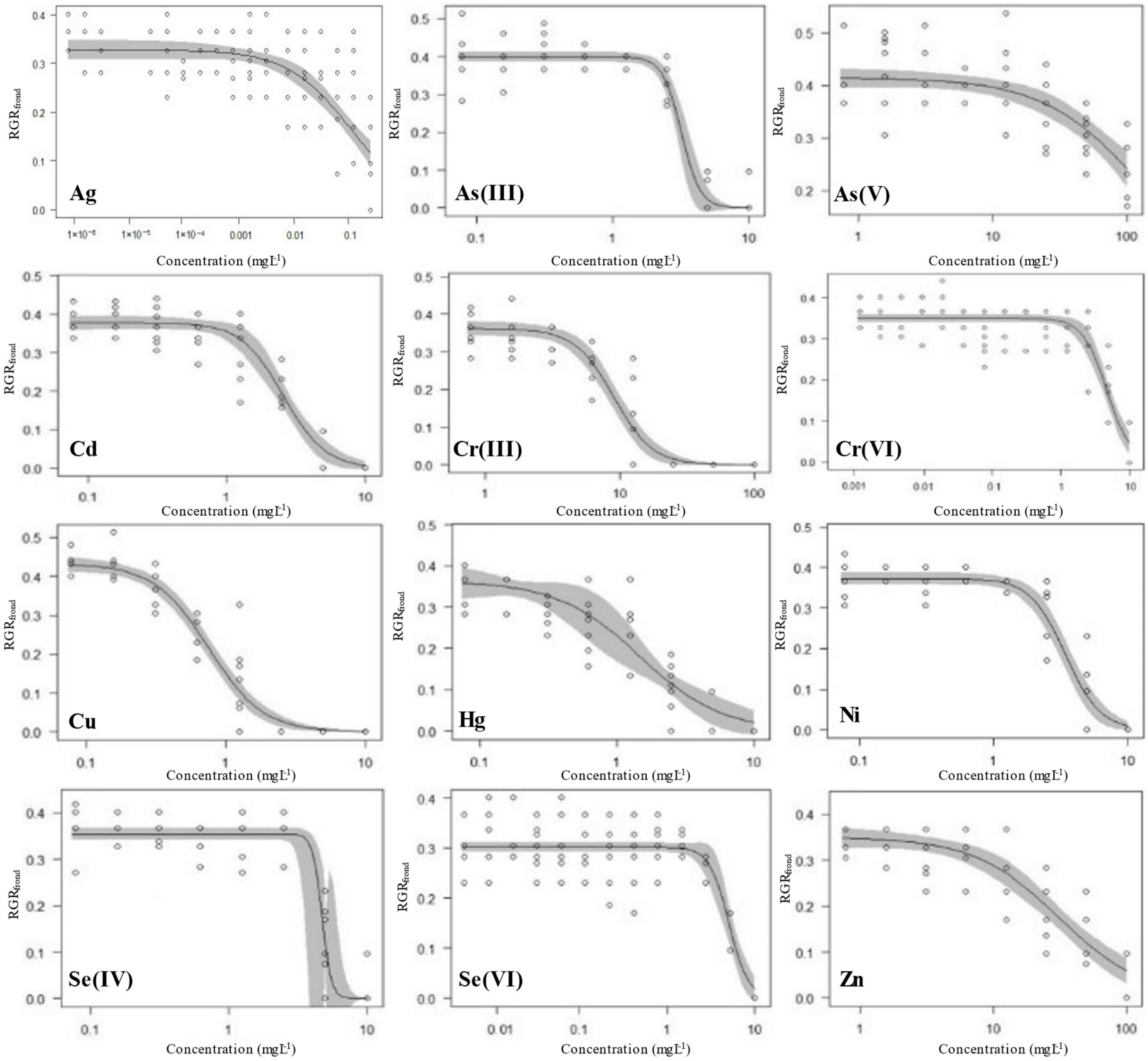

Supplement: Supplementary file 1 [file plants-13-00215-s001.zip › Figure S5-R1 v1.1.pdf]
