# Supplementary material for: Comparative Phytotoxicity of Metallic Elements on Duckweed Lemna gibba L. Using Growth- and Chlorophyll Fluorescence Induction-Based Endpoints
Source: Plants (Basel). 2024 Jan 12;13(2):215. doi: 10.3390/plants13020215 (PMC10821045; doi:10.3390/plants13020215)

Figure S7. 3-parameter log-logistic concentration-response model fittings of Y(II) for the tested metals and metalloids. Circles denote individual measurements, solid lines and shaded areas denote the fitted models and the corresponding standard errors of estimates.

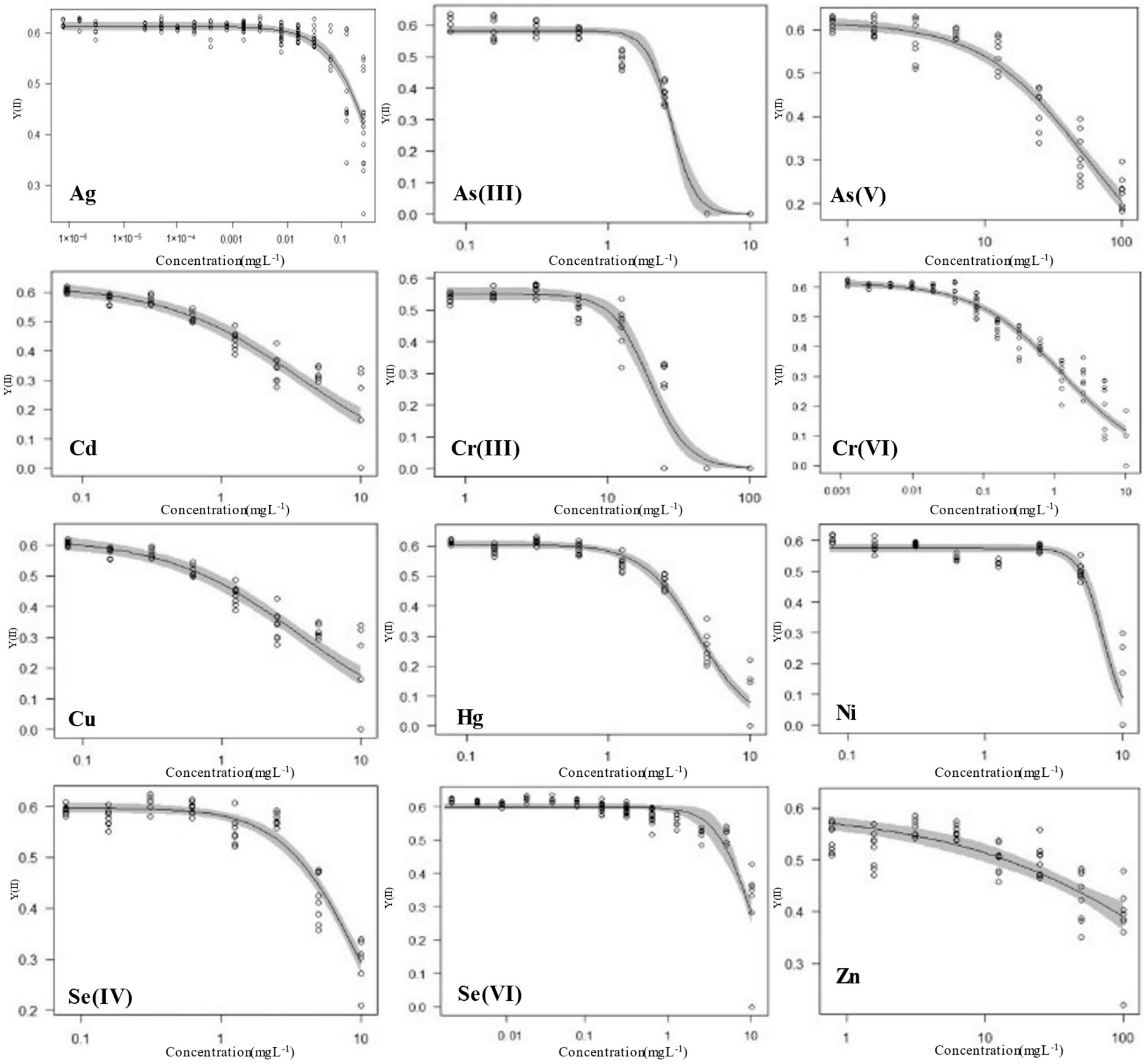

Supplement: Supplementary file 1 [file plants-13-00215-s001.zip › Figure S7-R1 v1.1.pdf]
