# Supplementary material for: Comparative Phytotoxicity of Metallic Elements on Duckweed Lemna gibba L. Using Growth- and Chlorophyll Fluorescence Induction-Based Endpoints
Source: Plants (Basel). 2024 Jan 12;13(2):215. doi: 10.3390/plants13020215 (PMC10821045; doi:10.3390/plants13020215)
Supplement: Supplementary file 1 [file plants-13-00215-s001.zip › File S1-R1.pdf]

Supplementary file

Comparative phytotoxicity of metallic elements on the duckweed *Lemna gibba* L. using growth- and chlorophyll fluorescence induction-based endpoints

Muhammad Irfan[1], Ilona Mészáros[1], Sándor Szabó[2], Viktor Oláh[1*]

This supplementary file consists of the studied species, parameters within the three endpoint-groups and the references used for the upset plot provided as **Figure. 1** in the main text of this study. We reviewed a total of 123 studies related to duckweed toxicity testing according to OECD (2006) [1] protocol. The following keywords were used in Scopus search engine, accessed on November 11, 2023:

"duckweed AND toxicity AND endpoint AND oecd AND PUBYEAR > 2012 AND PUBYEAR < 2024 AND (LIMIT-TO (DOCTYPE, "ar")) AND (LIMIT-TO (LANGUAGE, "English")) AND (LIMIT-TO (SRCTYPE,"j"))"

The results of this meta-analysis are based on 53 relevant studies [2–54] out of the total 123 studies found using the search keywords. The outcomes are provided as **Figure. 1** (part of the main text) and metadata figures (S1-S4; given below), while the references are provided at the end of this file.

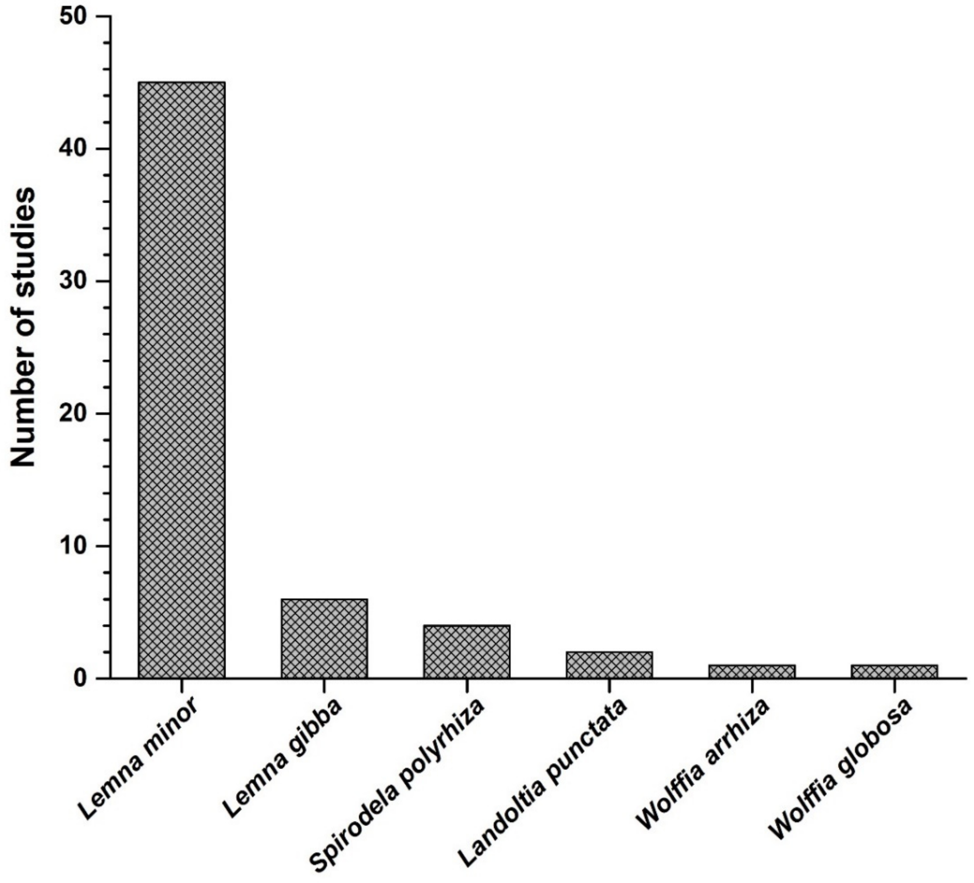

Metadata Figure S1. The occurrence of the studied duckweed species (*L. minor* [2–4,6–10,12–29,31–36,38–40,43,45–50,52–54], *L. gibba* [27,32,37,41,42,44], *S. polyrhiza* [5,9,17,36], *Land. Punctata* [30,51], *W. arrhiza* [9], *W. globosa* [11]) in different ecotoxicological studies conducted according to OECD (2006) [1] guidelines, using growth-, biochemistry- and ChlF-based endpoints.

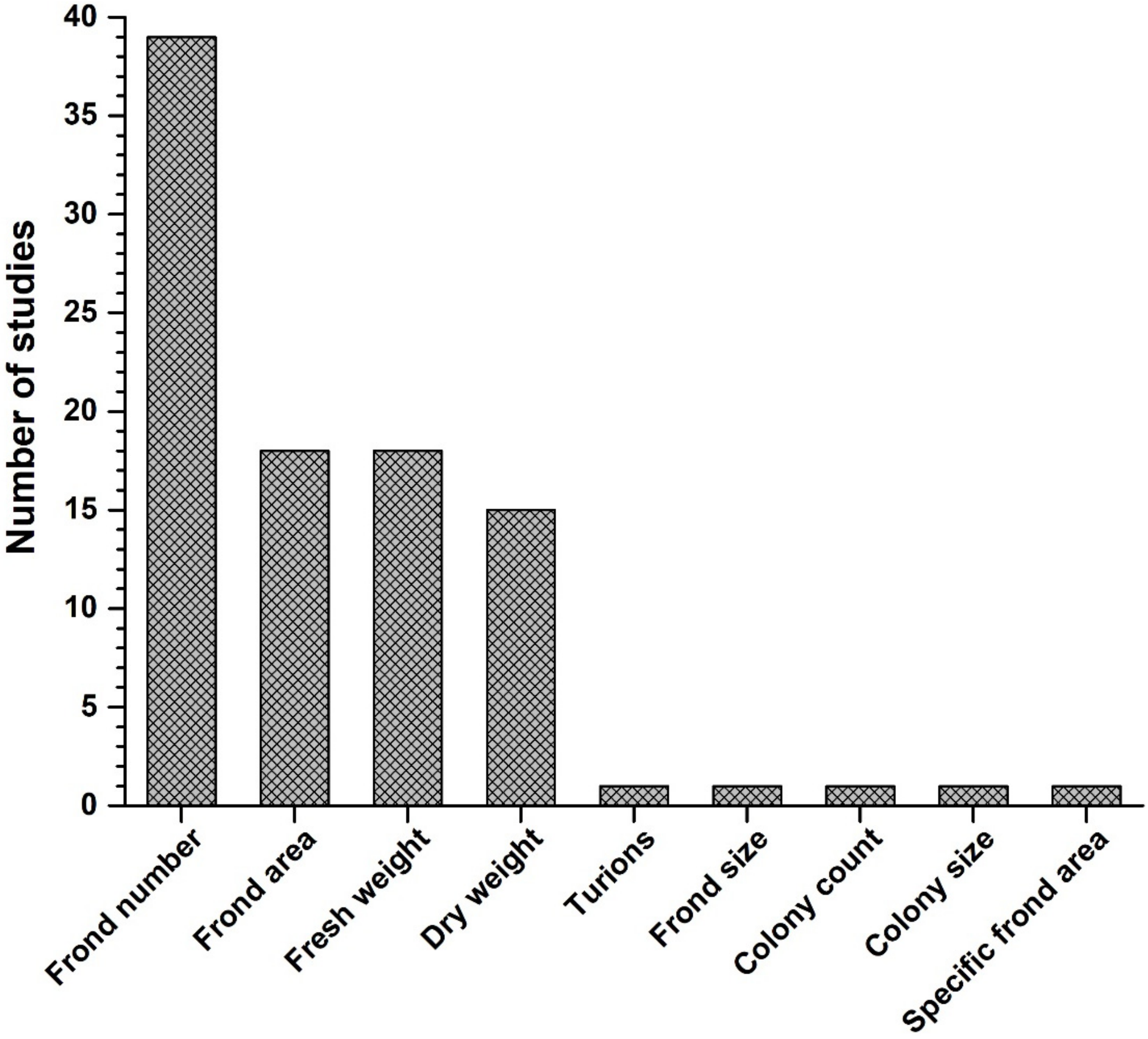

Metadata Figure S2. The occurrence of the studied growth and morphology-based test endpoints including frond number [1–6,9–13,15–22,24,26–29,31,32,34,35,37–47,49–54], frond area [5,8,9,12,17,19,22,23,30,34,36,41,42,46–48,52,54], fresh weight [13,14,23,25,29,31–33,39,40,42,45–47,50,52–54], dry weight [7,9,10,16,18,28,32,34,39,42,43,46,47,53,54], turions [5], frond size [42], colony count [32], colony size [40] and specific frond area [42] used in different ecotoxicological studies according to the OECD (2006) [1] guidelines.

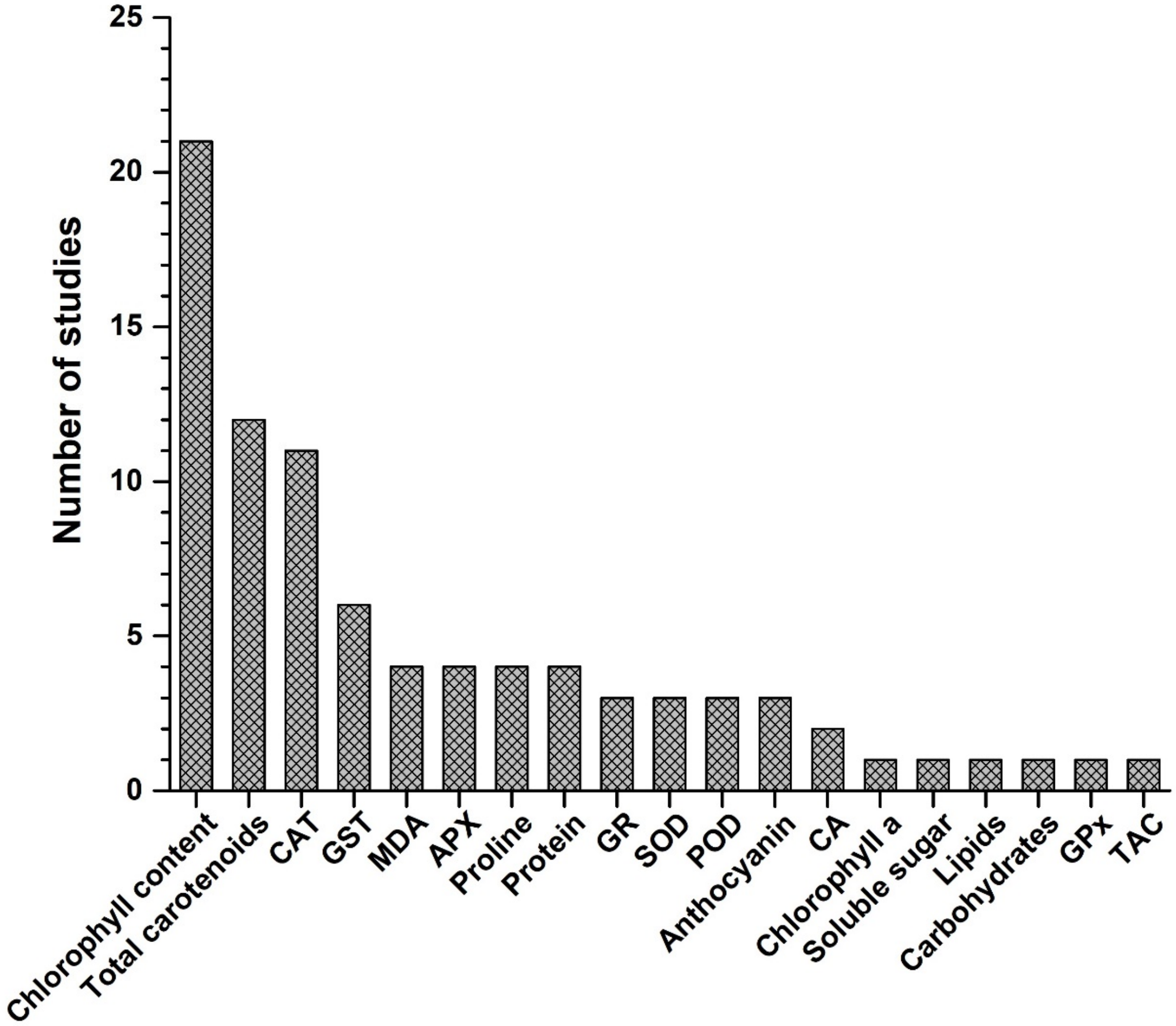

Metadata Figure S3. The occurrence of the studied biochemical markers as test endpoints including chlorophyll content [2–4,8,14,15,19,24–27,34,35,37–39,42,44,45,47,52], total carotenoids [8,24,26,27,34,37,38,42,44,45,47,52], catalase (CAT) [8,14,24,26,27,37,38,40,44,47,54], glutathione S-transferases (GST) [8,24,26,37,40,44], malondialdehyde (MDA) [2,3,14,34], ascorbate peroxidase (APx) [14,24,37,54], glutathione reductase (GR) [14,37,54], superoxide dismutase (SOD) [14,37,54], peroxidase (POD) [14,47,54], anthocyanin [27,38,42], carbonic anhydrase (CA) [26,44], chlorophyll a [22], soluble sugars [14], lipids [35], carbohydrates [35], glutathione peroxidase (GPx) [40] and total antioxidant capacity (TAC) [35] used in different ecotoxicological studies according to the OECD (2006) [1] guidelines.

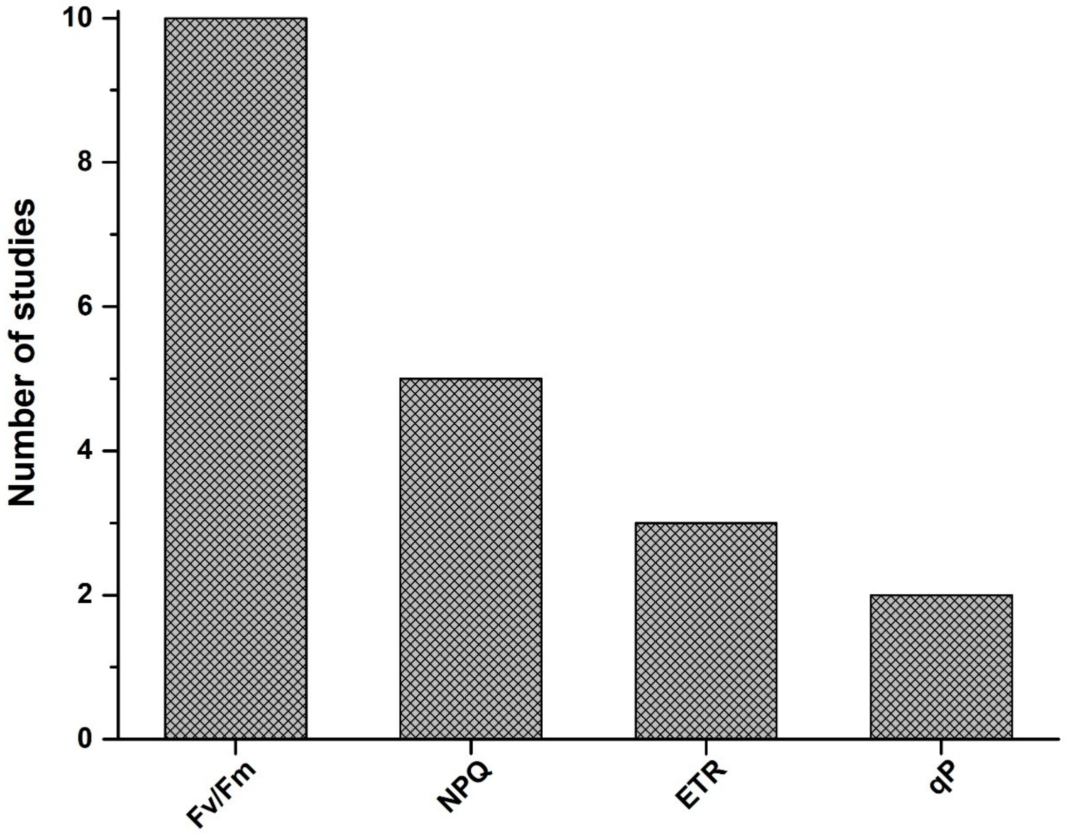

Metadata Figure S4. The frequency of the studied ChlF-based test endpoints used in different ecotoxicological studies according to the OECD (2006) [1] guidelines. The studied endpoints in this figure include F_v/F_m (denotes the maximal quantum yield of PSII in dark-adapted state) [10,13,14,17,27,34,46,47,52,53], NPQ (stands for non-photochemical quenching) [17,34,46,52,53], ETR (Electron transport rate) [17,34,46]and qP (photochemical quenching) [52,53].

References

1. OECD Guidelines for the Testing of Chemicals, Revised Proposal for a New Guideline 221, *Lemna* Sp. Growth Inhibition Test. **2006**.
2. Rodrigues, S.; Pinto, I.; Martins, F.; Formigo, N.; Antunes, S.C. An Ecotoxicological Approach Can Complement the Assessment of Natural Waters from Portuguese Reservoirs? *Environ. Sci. Pollut. Res. Int.* **2022**, *29*, 52147–52161. https://doi.org/10.1007/s11356-022-19504-4.
3. Diogo, B.S.; Rodrigues, S.; Lage, O.M.; Antunes, S.C. Are the Ecotoxicological Tools Viable to Evaluate the Effectiveness of Wastewater Treatment Plant Effluents? *Int. J. Environ. Sci. Technol.* **2023**, *20*, 11943–11962. https://doi.org/10.1007/s13762-023-04791-2.
4. Fekete-Kertész, I.; Kunglné-Nagy, Z.; Gruiz, K.; Magyar, Á.; Farkas, É.; Molnár, M. Assessing Toxicity of Organic Aquatic Micropollutants Based on the Total Chlorophyll Content of *Lemna minor* as a Sensitive Endpoint. *Period. Polytech. Chem. Eng.* **2015**, *59*, 262–271. https://doi.org/10.3311/PPch.8077.
5. Oláh, V.; Hepp, A.; Mészáros, I. Assessment of Giant Duckweed (*Spirodela polyrhiza* L. Schleiden) Turions as Model Objects in Ecotoxicological Applications. *Bull. Environ. Contam. Toxicol.* **2016**, *96*, 596–601. https://doi.org/10.1007/s00128-016-1765-z.
6. Boros, B.-V.; Dascalu, D.; Ostafe, V.; Isvoran, A. Assessment of the Effects of Chitosan, Chitooligosaccharides and Their Derivatives on *Lemna minor*. *Molecules* **2022**, *27*, 6123. https://doi.org/10.3390/molecules27186123.
7. Gavina, A.; Antunes, S.C.; Pinto, G.; Claro, M.T.; Santos, C.; Gonçalves, F.; Pereira, R. Can Physiological Endpoints Improve the Sensitivity of Assays with Plants in the Risk Assessment of Contaminated Soils? *PLOS ONE* **2013**, *8*, e59748. https://doi.org/10.1371/journal.pone.0059748.
8. Markovic, M.; Neale, P.A.; Nidumolu, B.; Kumar, A. Combined Toxicity of Therapeutic Pharmaceuticals to Duckweed, *Lemna minor*. *Ecotoxicol. Environ. Saf.* **2021**, *208*, 111428. https://doi.org/10.1016/j.ecoenv.2020.111428.
9. Song, L.; Vijver, M.G.; Peijnenburg, W.J.G.M. Comparative Toxicity of Copper Nanoparticles across Three Lemnaceae Species. *Sci. Total Environ.* **2015**, *518–519*, 217–224. https://doi.org/10.1016/j.scitotenv.2015.02.079.
10. Dalton, R.L.; Nussbaumer, C.; Pick, F.R.; Boutin, C. Comparing the Sensitivity of Geographically Distinct *Lemna minor* Populations to Atrazine. *Ecotoxicol. Lond. Engl.* **2013**, *22*, 718–730. https://doi.org/10.1007/s10646-013-1064-y.
11. Kurnia, K.A.; Lin, Y.-T.; Farhan, A.; Malhotra, N.; Luong, C.T.; Hung, C.-H.; Roldan, M.J.M.; Tsao, C.-C.; Cheng, T.-S.; Hsiao, C.-D. Deep Learning-Based Automatic Duckweed Counting Using StarDist and Its Application on Measuring Growth Inhibition Potential of Rare Earth Elements as Contaminants of Emerging Concerns. *Toxics* **2023**, *11*, 680. https://doi.org/10.3390/toxics11080680.
12. Van Antro, M.; Prelovsek, S.; Ivanovic, S.; Gawehns, F.; Wagemaker, N.C.A.M.; Mysara, M.; Horemans, N.; Vergeer, P.; Verhoeven, K.J.F. DNA Methylation in Clonal Duckweed (*Lemna minor* L.) Lineages Reflects Current and Historical Environmental Exposures. *Mol. Ecol.* **2023**, *32*, 428–443. https://doi.org/10.1111/mec.16757.
13. Roubeau Dumont, E.; Larue, C.; Lorber, S.; Gryta, H.; Billoir, E.; Gross, E.M.; Elger, A. Does Intraspecific Variability Matter in Ecological Risk Assessment? Investigation of Genotypic Variations in Three Macrophyte Species Exposed to Copper. *Aquat. Toxicol.* **2019**, *211*, 29–37. https://doi.org/10.1016/j.aquatox.2019.03.012.
14. Liang, J.; Li, Y.; Xie, P.; Liu, C.; Yu, L.; Ma, X. Dualistic Effects of Bisphenol A on Growth, Photosynthetic and Oxidative Stress of Duckweed (*Lemna minor*). *Environ. Sci. Pollut. Res. Int.* **2022**, *29*, 87717–87729. https://doi.org/10.1007/s11356-022-21785-8.
15. Fekete-Kertész, I.; Stirling, T.; Vaszita, E.; Berkl, Z.; Farkas, É.; Hedwig, S.; Remmen, K.; Lenz, M.; Molnár, M.; Feigl, V. Ecotoxicity Attenuation by Acid-Resistant Nanofiltration in Scandium Recovery from TiO_2 Production Waste. *Heliyon* **2023**, *9*, e15512. https://doi.org/10.1016/j.heliyon.2023.e15512.
16. Andreani, T.; Nogueira, V.; Gavina, A.; Fernandes, S.; Rodrigues, J.L.; Pinto, V.V.; Ferreira, M.J.; Silva, A.M.; Pereira, C.M.; Pereira, R. Ecotoxicity to Freshwater Organisms and Cytotoxicity of Nanomaterials: Are We Generating Sufficient Data for Their Risk Assessment? *Nanomaterials* **2021**, *11*, 66. https://doi.org/10.3390/nano11010066.
17. Pietrini, F.; Iannilli, V.; Passatore, L.; Carloni, S.; Sciacca, G.; Cerasa, M.; Zacchini, M. Ecotoxicological and Genotoxic Effects of Dimethyl Phthalate (DMP) on *Lemna Minor* L. and *Spirodela polyrhiza* (L.) Schleid. Plants under a Short-Term Laboratory Assay. *Sci. Total Environ.* **2022**, *806*, 150972. https://doi.org/10.1016/j.scitotenv.2021.150972.
18. Groth, V.A.; Carvalho-Pereira, T.; da Silva, E.M.; Niemeyer, J.C. Ecotoxicological Assessment of Biosolids by Microcosms. *Chemosphere* **2016**, *161*, 342–348. https://doi.org/10.1016/j.chemosphere.2016.07.029.
19. Sackey, L.N.A.; Mocová, K.A.; Kočí, V. Ecotoxicological Effect of Aged Wood Leachates to Aquatic Organisms. *Water* **2020**, *12*, 2091. https://doi.org/10.3390/w12082091.
20. Godoy, A.A.; Kummrow, F.; Pamplin, P.A.Z. Ecotoxicological Evaluation of Propranolol Hydrochloride and Losartan Potassium to *Lemna minor* L. (1753) Individually and in Binary Mixtures. *Ecotoxicology* **2015**, *24*, 1112–1123. https://doi.org/10.1007/s10646-015-1455-3.
21. Soares, C.; Fernandes, B.; Paiva, C.; Nogueira, V.; Cachada, A.; Fidalgo, F.; Pereira, R. Ecotoxicological Relevance of Glyphosate and Flazasulfuron to Soil Habitat and Retention Functions – Single vs Combined Exposures. *J. Hazard. Mater.* **2023**, *442*, 130128. https://doi.org/10.1016/j.jhazmat.2022.130128.
22. Ikebe Otomo, J.; Araujo de Jesus, T.; Gomes Coelho, L.H.; Rebelo Monteiro, L.; Hunter, C.; Helwig, K.; Roberts, J.; Pahl, O. Effect of Eight Common Brazilian Drugs on *Lemna minor* and *Salvinia auriculata* Growth. *Environ. Sci. Pollut. Res. Int.* **2021**, *28*, 43747–43762. https://doi.org/10.1007/s11356-021-13795-9.

23. Hlavkova, D.; Caloudova, H.; Palikova, P.; Kopel, P.; Plhalova, L.; Beklova, M.; Havelkova, B. Effect of Gold Nanoparticles and Ions Exposure on the Aquatic Organisms. *Bull. Environ. Contam. Toxicol.* **2020**, *105*, 530–537. https://doi.org/10.1007/s00128-020-02988-6.
24. Alkimin, G.D.; Daniel, D.; Dionísio, R.; Soares, A.M.V.M.; Barata, C.; Nunes, B. Effects of Diclofenac and Salicylic Acid Exposure on *Lemna minor*: Is Time a Factor? *Environ. Res.* **2019**, *177*, 108609. https://doi.org/10.1016/j.envres.2019.108609.
25. Castro, V.L.; Jonsson, C.M.; Silva, M.S.G.M.; Castanha, R.; Vallim, J.H.; da Silva, L.A.G.; de Oliveira, R.M.D.; Correa, D.S.; Ferreira, M.D. Estimates of AgNP Toxicity Thresholds in Support of Environmental Safety Policies. *J. Nanoparticle Res.* **2022**, *24*, 9. https://doi.org/10.1007/s11051-021-05357-4.
26. Alkimin, G.D.; Soares, A.M.V.M.; Barata, C.; Nunes, B. Evaluation of Ketoprofen Toxicity in Two Freshwater Species: Effects on Biochemical, Physiological and Population Endpoints. *Environ. Pollut.* **2020**, *265*, 114993. https://doi.org/10.1016/j.envpol.2020.114993.
27. Alkimin, G.D.; Daniel, D.; Frankenbach, S.; Serôdio, J.; Soares, A.M.V.M.; Barata, C.; Nunes, B. Evaluation of Pharmaceutical Toxic Effects of Non-Standard Endpoints on the Macrophyte Species *Lemna minor* and *Lemna gibba*. *Sci. Total Environ.* **2019**, *657*, 926–937. https://doi.org/10.1016/j.scitotenv.2018.12.002.
28. Castro, A.M.; Nogueira, V.; Lopes, I.; Rocha-Santos, T.; Pereira, R. Evaluation of the Potential Toxicity of Effluents from the Textile Industry before and after Treatment. *Appl. Sci.* **2019**, *9*, 3804. https://doi.org/10.3390/app9183804.
29. Knežević, V.; Tunić, T.; Gajić, P.; Marjan, P.; Savić, D.; Tenji, D.; Teodorović, I. Getting More Ecologically Relevant Information from Laboratory Tests: Recovery of *Lemna minor* After Exposure to Herbicides and Their Mixtures. *Arch. Environ. Contam. Toxicol.* **2016**, *71*, 572–588. https://doi.org/10.1007/s00244-016-0321-5.
30. Mohr, S.; Schott, J.; Hoenemann, L.; Feibicke, M. *Glyceria maxima* as New Test Species for the EU Risk Assessment for Herbicides: A Microcosm Study. *Ecotoxicol. Lond. Engl.* **2015**, *24*, 309–320. https://doi.org/10.1007/s10646-014-1379-3.
31. Magahud, J.C.; Dalumpines, S.L.P. Growth of Duckweeds (*Lemna minor* L.) as Affected by Light Intensity, Nutrient Solution Concentration, and Light x Nutrient Interaction. **2021**, *14*.
32. Burns, M.; Hanson, M.L.; Prosser, R.S.; Crossan, A.N.; Kennedy, I.R. Growth Recovery of *Lemna gibba* and *Lemna minor* Following a 7-Day Exposure to the Herbicide Diuron. *Bull. Environ. Contam. Toxicol.* **2015**, *95*, 150–156. https://doi.org/10.1007/s00128-015-1575-8.
33. Havelkova, B.; Hlavkova, D.; Kovacova, V.; Beklova, M. Herbicides in the Cave Environment: Ecotoxicological Risks. *Fresenius Environ. Bull.* **2019**, *28*, 781–786.
34. Xie, L.; Gomes, T.; Solhaug, K.A.; Song, Y.; Tollefsen, K.E. Linking Mode of Action of the Model Respiratory and Photosynthesis Uncoupler 3,5-Dichlorophenol to Adverse Outcomes in *Lemna minor*. *Aquat. Toxicol. Amst. Neth.* **2018**, *197*, 98–108. https://doi.org/10.1016/j.aquatox.2018.02.005.
35. Rozman, U.; Jemec Kokalj, A.; Dolar, A.; Drobne, D.; Kalčíková, G. Long-Term Interactions between Microplastics and Floating Macrophyte *Lemna minor*: The Potential for Phytoremediation of Microplastics in the Aquatic Environment. *Sci. Total Environ.* **2022**, *831*, 154866. https://doi.org/10.1016/j.scitotenv.2022.154866.
36. Tofan, L.; Niță, V.; Nenciu, M.; Coatu, V.; Lazăr, L.; Damir, N.; Vasile, D.; Popoviciu, D.R.; Brotea, A.-G.; Curtean-Bănăduc, A.M.; et al. Multiple Assays on Non-Target Organisms to Determine the Risk of Acute Environmental Toxicity in Tebuconazole-Based Fungicides Widely Used in the Black Sea Coastal Area. *Toxics* **2023**, *11*, 597. https://doi.org/10.3390/toxics11070597.
37. Varga, M.; Horvatić, J.; Barišić, L.; Lončarić, Z.; Dutour Sikirić, M.; Erceg, I.; Kočić, A.; Štolfa Čamagajevac, I. Physiological and Biochemical Effect of Silver on the Aquatic Plant *Lemna gibba* L.: Evaluation of Commercially Available Product Containing Colloidal Silver. *Aquat. Toxicol. Amst. Neth.* **2019**, *207*, 52–62. https://doi.org/10.1016/j.aquatox.2018.11.018.
38. de Alkimin, G.D.; Paisio, C.; Agostini, E.; Nunes, B. Phytoremediation Processes of Domestic and Textile Effluents: Evaluation of the Efficacy and Toxicological Effects in *Lemna minor* and *Daphnia magna*. *Environ. Sci. Pollut. Res. Int.* **2020**, *27*, 4423–4441. https://doi.org/10.1007/s11356-019-07098-3.
39. Logeshwaran, P.; Sivaram, A.K.; Yadav, M.; Chadalavada, S.; Naidu, R.; Megharaj, M. Phytotoxicity of Class B Aqueous Firefighting Formulations, Tridol S 3 and 6% to *Lemna minor*. *Environ. Technol. Innov.* **2020**, *18*, 100688. https://doi.org/10.1016/j.eti.2020.100688.
40. Pereira, S.P.P.; Jesus, F.; Aguiar, S.; de Oliveira, R.; Fernandes, M.; Ranville, J.; Nogueira, A.J.A. Phytotoxicity of Silver Nanoparticles to *Lemna minor*: Surface Coating and Exposure Period-Related Effects. *Sci. Total Environ.* **2018**, *618*, 1389–1399. https://doi.org/10.1016/j.scitotenv.2017.09.275.
41. Mohiley, A.; Franzaring, J.; Calvo, O.C.; Fangmeier, A. Potential Toxic Effects of Aircraft De-Icers and Wastewater Samples Containing These Compounds. *Environ. Sci. Pollut. Res. Int.* **2015**, *22*, 13094–13101. https://doi.org/10.1007/s11356-015-4358-1.
42. Di Baccio, D.; Pietrini, F.; Bertolotto, P.; Pérez, S.; Barcelò, D.; Zacchini, M.; Donati, E. Response of *Lemna gibba* L. to High and Environmentally Relevant Concentrations of Ibuprofen: Removal, Metabolism and Morpho-Physiological Traits for Biomonitoring of Emerging Contaminants. *Sci. Total Environ.* **2017**, *584–585*, 363–373. https://doi.org/10.1016/j.scitotenv.2016.12.191.
43. Gopalapillai, Y.; Vigneault, B.; Hale, B.A. Root Length of Aquatic Plant, *Lemna minor* L., as an Optimal Toxicity Endpoint for Biomonitoring of Mining Effluents. *Integr. Environ. Assess. Manag.* **2014**, *10*, 493–497. https://doi.org/10.1002/ieam.1558.

44. Daniel, D.; de Alkimin, G.D.; Nunes, B. Single and Combined Effects of the Drugs Salicylic Acid and Acetazolamide: Adverse Changes in Physiological Parameters of the Freshwater Macrophyte, *Lemna gibba*. *Environ. Toxicol. Pharmacol.* **2020**, *79*, 103431. https://doi.org/10.1016/j.etap.2020.103431.
45. Gabriel, A.; Venâncio, C.; Sousa, J.P.; Leston, S.; Ramos, F.; Soares, A.M.V.M.; Lopes, I. Soil pH Matters in the Ecotoxicity of Basamid® to Freshwater Microalgae and Macrophytes. *Sci. Total Environ.* **2023**, *859*, 160165. https://doi.org/10.1016/j.scitotenv.2022.160165.
46. Grenni, P.; Patrolecco, L.; Rauseo, J.; Spataro, F.; Di Lenola, M.; Aimola, G.; Zacchini, M.; Pietrini, F.; Di Baccio, D.; Stanton, I.C.; et al. Sulfamethoxazole Persistence in a River Water Ecosystem and Its Effects on the Natural Microbial Community and *Lemna minor* Plant. *Microchem. J.* **2019**, *149*, 103999. https://doi.org/10.1016/j.microc.2019.103999.
47. Sikorski, Ł.; Baciak, M.; Bęś, A.; Adomas, B. The Effects of Glyphosate-Based Herbicide Formulations on *Lemna minor*, a Non-Target Species. *Aquat. Toxicol. Amst. Neth.* **2019**, *209*, 70–80. https://doi.org/10.1016/j.aquatox.2019.01.021.
48. Modlitbová, P.; Hlaváček, A.; Švestková, T.; Pořízka, P.; Šimoníková, L.; Novotný, K.; Kaiser, J. The Effects of Photon-Upconversion Nanoparticles on the Growth of Radish and Duckweed: Bioaccumulation, Imaging, and Spectroscopic Studies. *Chemosphere* **2019**, *225*, 723–734. https://doi.org/10.1016/j.chemosphere.2019.03.074.
49. Kokalj, A.J.; Novak, S.; Talaber, I.; Kononenko, V.; Mali, L.B.; Vodovnik, M.; Žegura, B.; Eleršek, T.; Kalčikova, G.; Gotvajn, A.Ž.; et al. The First Comprehensive Safety Study of Magnéli Phase Titanium Suboxides Reveals No Acute Environmental Hazard. *Environ. Sci. Nano* **2019**, *6*, 1131–1139. https://doi.org/10.1039/C8EN01119B.
50. Mihajlović, V.; Tomić, T.; Tubić, A.; Molnar Jazić, J.; Ivančev Tumbas, I.; Šunjka, D.; Lazić, S.; Teodorović, I. The Impact of Humic Acid on Toxicity of Individual Herbicides and Their Mixtures to Aquatic Macrophytes. *Environ. Sci. Pollut. Res. Int.* **2019**, *26*, 23571–23582. https://doi.org/10.1007/s11356-019-05629-6.
51. Facin, F.; de Melo, J.V.S.; Lalau, C.M.; Nogueira, D.J.; Puerari, R.C.; Matias, W.G. Toxicological Effects of Leachate Extracts from Asphalt Mixtures Nanomodified under *Daphnia magna* and *Landoltia punctata* Test Organisms. *Chemosphere* **2021**, *285*, 131463. https://doi.org/10.1016/j.chemosphere.2021.131463.
52. Xie, L.; Song, Y.; Petersen, K.; Solhaug, K.A.; Lind, O.C.; Brede, D.A.; Salbu, B.; Tollefsen, K.E. Ultraviolet B Modulates Gamma Radiation-Induced Stress Responses in *Lemna minor* at Multiple Levels of Biological Organisation. *Sci. Total Environ.* **2022**, *846*, 157457. https://doi.org/10.1016/j.scitotenv.2022.157457.
53. Roubeau Dumont, E.; Gao, X.; Zheng, J.; Macairan, J.; Hernandez, L.M.; Baesu, A.; Bayen, S.; Robinson, S.A.; Ghoshal, S.; Tufenkji, N. Unraveling the Toxicity of Tire Wear Contamination in Three Freshwater Species: From Chemical Mixture to Nanoparticles. *J. Hazard. Mater.* **2023**, *453*, 131402. https://doi.org/10.1016/j.jhazmat.2023.131402.
54. Van Hoeck, A.; Horemans, N.; Van Hees, M.; Nauts, R.; Knapen, D.; Vandenhove, H.; Blust, R. β-Radiation Stress Responses on Growth and Antioxidative Defense System in Plants: A Study with Strontium-90 in *Lemna minor*. *Int. J. Mol. Sci.* **2015**, *16*, 15309–15327. https://doi.org/10.3390/ijms160715309.
