# Supplementary material for: Comparative Phytotoxicity of Metallic Elements on Duckweed Lemna gibba L. Using Growth- and Chlorophyll Fluorescence Induction-Based Endpoints
Source: Plants (Basel). 2024 Jan 12;13(2):215. doi: 10.3390/plants13020215 (PMC10821045; doi:10.3390/plants13020215)
Supplement: Supplementary file 1 [file plants-13-00215-s001.zip › Table S1-R1.pdf]

**Table S1.** The results of the 3-parameter log-logistic model fittings (DF – degrees of freedom, RSS - residual sum of squares, F-value, p-value and Pseudo $R^2$), and the calculated 20% ($EC_{20}$) and 50% effective concentrations ($EC_{50}$) for frond number- ($RGR_{frond}$) and frond area-based relative growth rates ($RGR_{area}$), dark adapted- ($F_v/F_o$) and light adapted photochemical efficiency (Y(II)) as estimates ± standard errors of estimates.

| Toxicant | Parameter | DF | RSS | F-value | p-value | Pseudo $R^2$ | EC20 (mg $L^{-1}$ ±SE) | EC50 (mg $L^{-1}$ ±SE) |
|---|---|---|---|---|---|---|---|---|
| **Ag** | $RGR_{frond}$ | 169 | 0.69 | 2.62 | <0.005 | 0.76 | 0.016±0.003 | 0.11±0.02 |
| | $RGR_{area}$ | 169 | 0.26 | 14.94 | <0.001 | 0.92 | 0.0001±0.00006 | 0.005±0.001 |
| | $F_v/F_o$ | 169 | 5.27 | 1.18 | 0.300 | 0.71 | 0.31±0.03* | 1.27±0.43* |
| | Y(II) | 169 | 0.23 | 0.31 | 0.990 | 0.85 | 0.14±0.01* | 0.55±0.07* |
| **As(III)** | $RGR_{frond}$ | 69 | 0.15 | 0.68 | 0.670 | 0.96 | 2.59±0.12 | 3.21±0.17 |
| | $RGR_{area}$ | 69 | 0.19 | 2.94 | 0.010 | 0.94 | 0.90±0.12 | 1.77±0.15 |
| | $F_v/F_o$ | 69 | 2.85 | 1.22 | 0.310 | 0.99 | 0.93±0.05 | 1.84±0.06 |
| | Y(II) | 69 | 0.11 | 25.55 | <0.001 | 0.99 | 2.14±0.10 | 2.80±0.06 |
| **As(V)** | $RGR_{frond}$ | 69 | 0.20 | 0.40 | 0.880 | 0.73 | 41.43±8.07 | 132.18±23.91* |
| | $RGR_{area}$ | 69 | 0.11 | 0.95 | 0.460 | 0.91 | 24.75±3.02 | 60.70±4.41 |
| | $F_v/F_o$ | 69 | 4.50 | 1.08 | 0.380 | 0.93 | 14.41±1.62 | 47.15±2.68 |
| | Y(II) | 69 | 0.10 | 2.55 | 0.030 | 0.96 | 16.10±1.61 | 54.33±3.01 |
| **Cd** | $RGR_{frond}$ | 69 | 0.17 | 2.97 | 0.010 | 0.95 | 1.59±0.22 | 2.58±0.17 |
| | $RGR_{area}$ | 69 | 1.02 | 0.82 | 0.560 | 0.97 | 0.44±0.04 | 0.73±0.04 |
| | $F_v/F_o$ | 69 | 8.12 | 11.05 | <0.001 | 0.96 | 0.54±0.11 | 2.45±0.25 |
| | Y(II) | 69 | 0.25 | 2.47 | 0.030 | 0.93 | 0.78±0.17 | 3.59±0.38 |
| **Cr(III)** | $RGR_{frond}$ | 69 | 0.17 | 0.66 | 0.680 | 0.96 | 5.68±0.61 | 9.02±0.60 |
| | $RGR_{area}$ | 69 | 0.05 | 1.05 | 0.400 | 0.98 | 4.21±0.31 | 6.22±0.23 |
| | $F_v/F_o$ | 69 | 8.72 | 6.01 | <0.001 | 0.96 | 7.52±0.80 | 12.35±0.74 |
| | Y(II) | 69 | 0.24 | 1.34 | 0.250 | 0.97 | 12.92±1.11 | 19.92±0.99 |
| **Cr(VI)** | $RGR_{frond}$ | 132 | 0.32 | 4.60 | <0.001 | 0.87 | 2.69±0.36 | 4.69±0.32 |
| | $RGR_{area}$ | 132 | 0.34 | 22.01 | <0.001 | 0.89 | 0.02±0.002 | 0.66±0.15 |
| | $F_v/F_o$ | 132 | 6.98 | 10.01 | <0.001 | 0.98 | 0.09±0.008 | 0.42±0.02 |
| | Y(II) | 132 | 0.27 | 6.87 | <0.001 | 0.97 | 0.18±0.03 | 1.28±0.1 |

| | | | | | | | | |
|---|---|---|---|---|---|---|---|---|
| **Cu** | $RGR_{frond}$ | 69 | 0.14 | 1.18 | 0.330 | 0.97 | 0.40±0.04 | 0.75±0.04 |
| | $RGR_{area}$ | 69 | 0.03 | 1.75 | 0.120 | 0.99 | 0.29±0.01 | 0.37±0.01 |
| | $F_v/F_o$ | 69 | 4.36 | 22.54 | <0.001 | 0.98 | 0.45±0.05 | 1.23±0.08 |
| | Y(II) | 69 | 0.21 | 11.42 | <0.001 | 0.97 | 0.90±0.10 | 1.86±0.11 |
| **Hg** | $RGR_{frond}$ | 69 | 0.26 | 9.24 | <0.001 | 0.92 | 0.56±0.33 | 1.47±0.38 |
| | $RGR_{area}$ | 69 | 0.10 | 9.31 | <0.001 | 0.96 | 0.05±0.01 | 0.24±0.03 |
| | $F_v/F_o$ | 69 | 3.72 | 1.58 | 0.170 | 0.97 | 2.60±0.14 | 4.87±0.17 |
| | Y(II) | 69 | 0.11 | 2.05 | 0.070 | 0.98 | 2.32±0.15 | 4.30±0.15 |
| **Ni** | $RGR_{frond}$ | 69 | 0.17 | 0.21 | 0.970 | 0.94 | 2.27±0.20 | 3.44±0.20 |
| | $RGR_{area}$ | 69 | 0.08 | 2.43 | 0.030 | 0.96 | 1.77±0.20 | 3.28±0.20 |
| | $F_v/F_o$ | 69 | 6.36 | 28.93 | <0.001 | 0.94 | 2.73±0.47 | 5.81±0.35 |
| | Y(II) | 69 | 0.16 | 2.80 | 0.010 | 0.95 | 5.51±0.26 | 7.21±0.25 |
| **Se(IV)** | $RGR_{frond}$ | 69 | 0.16 | 1.00 | 0.430 | 0.93 | 4.27±1.68 | 4.73±0.64 |
| | $RGR_{area}$ | 69 | 1.36 | 15.4 | <0.001 | 0.90 | 4.01±1.87 | 4.41±1.18 |
| | $F_v/F_o$ | 69 | 4.20 | 0.31 | 0.930 | 0.96 | 4.62±0.17 | 6.48±0.19 |
| | Y(II) | 69 | 0.06 | 6.56 | <0.001 | 0.95 | 4.10±0.25 | 9.73±0.42 |
| **Se(VI)** | $RGR_{frond}$ | 131 | 0.34 | 2.16 | 0.020 | 0.84 | 3.26±0.47 | 4.78±0.35 |
| | $RGR_{area}$ | 131 | 0.48 | 12.18 | <0.001 | 0.80 | 0.26±0.15 | 1.73±0.42 |
| | $F_v/F_o$ | 131 | 5.06 | 0.46 | 0.920 | 0.91 | 5.96±0.32 | 10.39±0.34* |
| | Y(II) | 131 | 0.29 | 3.24 | <0.001 | 0.87 | 4.83±0.59 | 9.40±0.47 |
| **Zn** | $RGR_{frond}$ | 69 | 0.21 | 1.76 | 0.120 | 0.89 | 11.28±2.93 | 31.23±3.90 |
| | $RGR_{area}$ | 69 | 0.10 | 7.37 | <0.001 | 0.93 | 0.27±0.10 | 3.88±0.78 |
| | $F_v/F_o$ | 69 | 4.78 | 13.25 | <0.001 | 0.96 | 24.42±2.41 | 45.31±2.28 |
| | Y(II) | 69 | 0.12 | 4.46 | <0.001 | 0.84 | 24.85±9.53 | 350.56±128.85* |

* Extrapolated values from the model fittings when the calculated effective concentrations were out of the applied concentration range.
